# An unconventional interaction between Dis1/TOG and Mal3/EB1 in fission yeast promotes the fidelity of chromosome segregation

**DOI:** 10.1242/jcs.197533

**Published:** 2016-12-15

**Authors:** Yuzy Matsuo, Sebastian P. Maurer, Masashi Yukawa, Silva Zakian, Martin R. Singleton, Thomas Surrey, Takashi Toda

**Affiliations:** 1 Synthetic and Systems Biochemistry of the Microtubule Cytoskeleton Laboratory, The Francis Crick Institute, 1 Midland Road, London NW1 1AT, UK; 2 Cell Regulation Laboratory, The Francis Crick Institute, 44 Lincoln's Inn Fields, London WC2A 3LY, UK; 3 Cell and Developmental Biology, Centre for Genomic Regulation (CRG), Barcelona Institute of Science and Technology (BIST), Dr. Aiguader 88, Barcelona 08003, Spain; 4 Universitat Pompeu Fabra (UPF), Barcelona 08002, Spain; 5 Hiroshima Research Center for Healthy Aging (HiHA), Department of Molecular Biotechnology, Graduate School of Advanced Science of Matter, Hiroshima University, 1-3-1 Kagamiyama, Higashi-Hiroshima 739-8530, Japan; 6 Structural Biology of Chromosome Segregation Laboratory, The Francis Crick Institute, 1 Midland Road, London NW1 1AT, UK

**Keywords:** Crystallography, EB1, Microtubule polymerase, TIRF microscopy, XMAP215, TOG

## Abstract

Dynamic microtubule plus-ends interact with various intracellular target regions such as the cell cortex and the kinetochore. Two conserved families of microtubule plus-end-tracking proteins, the XMAP215, ch-TOG or CKAP5 family and the end-binding 1 (EB1, also known as MAPRE1) family, play pivotal roles in regulating microtubule dynamics. Here, we study the functional interplay between fission yeast Dis1, a member of the XMAP215/TOG family, and Mal3, an EB1 protein. Using an *in vitro* microscopy assay, we find that purified Dis1 autonomously tracks growing microtubule ends and is a bona fide microtubule polymerase. Mal3 recruits additional Dis1 to microtubule ends, explaining the synergistic enhancement of microtubule dynamicity by these proteins. A non-canonical binding motif in Dis1 mediates the interaction with Mal3. X-ray crystallography shows that this new motif interacts in an unconventional configuration with the conserved hydrophobic cavity formed within the Mal3 C-terminal region that typically interacts with the canonical SXIP motif. Selectively perturbing the Mal3–Dis1 interaction in living cells demonstrates that it is important for accurate chromosome segregation. Whereas, in some metazoans, the interaction between EB1 and the XMAP215/TOG family members requires an additional binding partner, fission yeast relies on a direct interaction, indicating evolutionary plasticity of this critical interaction module.

## Abstract (precis)

**Summary:** We show that Dis1 and Mal3, fission yeast XMAP215/TOG and EB1 family members, directly interact in a non-canonical manner, thereby synergistically enhancing microtubule dynamics.

## INTRODUCTION

Microtubules (MTs) are structurally polar and highly dynamic tubulin polymers that undergo spontaneous transitions from growing to shrinking phases, known as dynamic instability (Mitchison and Kirschner, 1984). Such dynamic properties of MTs play an essential role in many cellular processes including intracellular transport, cell polarity and cell division (Desai and Mitchison, 1997). MT dynamics are regulated by a cohort of evolutionarily conserved MT-associated proteins (MAPs) (Akhmanova and Steinmetz, 2008). A subclass of MAPs, MT plus-end-tracking proteins (+TIPs) have unique properties, as they can specifically interact with the dynamic ends of MTs, thereby playing a decisive role in determining the characteristics of MT plus-ends in cells (Buey et al., 2012; Duellberg et al., 2013). In particular, end-binding 1 (EB1, also known as MAPRE1) and ch-TOG [known as XMAP215 (*Xenopus*) and CKAP5 (*Homo sapiens*); hereafter denoted TOG] are important +TIPs because they can both accumulate autonomously at MT ends independently of other MAPs, although by different mechanisms (Akhmanova and Hoogenraad, 2005).

EB1 family proteins bind in a nucleotide-dependent manner to an extended region at the growing MT end, resulting in a comet-like appearance at MT plus-ends in cells (Bieling et al., 2007, 2008; Maurer et al., 2011, 2012, 2014; Mohan et al., 2013; Zanic et al., 2009; Zhang et al., 2015). EB1 family proteins can have direct and indirect effects on the MT dynamics (Galjart, 2010); several *in vitro* experiments have suggested that purified EB1 family proteins promote the MT growth rate and simultaneously increase the catastrophe frequency (Bieling et al., 2007; Li et al., 2012; Vitre et al., 2008; Zanic et al., 2013).

*In vivo* EB1 family proteins recruit several other MAPs to MT plus-ends through direct protein–protein interactions. EB1 family proteins consist of four functional regions; the N-terminal calponin homology (CH) domain required for MT binding (Hayashi and Ikura, 2003), the medial coiled-coil region involved in homo-dimerisation (De Groot et al., 2010) followed by the EB homology (EBH) domain and finally the C-terminal EEY/F motif (Duellberg et al., 2013). The EBH domain specifically binds to an SXIP motif found in a variety of +TIPs (Buey et al., 2012; Duellberg et al., 2014; Honnappa et al., 2009), whereas the EEY/F motif at the C-terminus of EB1 family proteins binds to some CAP-Gly domains found in some MAPs (Duellberg et al., 2013; Honnappa et al., 2006; Weisbrich et al., 2007). MT plus-end recruitment of other +TIPs by EB1 family proteins is responsible for the indirect effects EB1 family proteins can have on MT behaviour, and hence on a variety of MT-dependent cellular processes.

Mal3, the sole EB1 homologue in fission yeast *Schizosaccharomyces pombe*, is nonessential for cell division, yet *mal3* deletion mutants display a variety of defects derived from abnormal MT architectures and dynamics. These include cell polarity defects during interphase (Beinhauer et al., 1997; Browning et al., 2003; Busch and Brunner, 2004; Busch et al., 2004) and chromosome segregation errors during mitosis (Asakawa et al., 2005, 2006; Asakawa and Toda, 2006; Beinhauer et al., 1997; Mana-Capelli et al., 2012). Mal3 has been shown to interact with the SXIP-motif- and CAP-Gly-domain-containing MAP Tip1, the fission yeast CLIP-170 orthologue, and the Tea2 kinesin, thereby playing a crucial role in regulation of interphase MT organisation and cell polarisation (Bieling et al., 2007; Browning et al., 2003; Busch et al., 2004). By contrast, our understanding of how Mal3 regulates mitotic progression remains poorly understood despite several earlier studies (Asakawa et al., 2006; Kerres et al., 2004). Work performed *in vitro* has indicated that Mal3 alone has some impact on MT dynamics (Bieling et al., 2007; des Georges et al., 2008; Katsuki et al., 2009); however, it is likely that Mal3 cooperates with other +TIPs during mitosis through direct interactions, as in interphase.

TOG proteins comprise another class of +TIPs that play pivotal roles in many MT-mediated processes (Al-Bassam and Chang, 2011; Kinoshita et al., 2002; Ohkura et al., 2001). Members of this protein family contain N-terminal TOG domains that bind soluble tubulin and a separate MT-binding site (Al-Bassam et al., 2006; Widlund et al., 2011) that, in combination, allow them to act as MT polymerases accelerating MT growth (Al-Bassam et al., 2012; Ayaz et al., 2012, 2014; Brouhard et al., 2008; Li et al., 2012; Podolski et al., 2014; Reber et al., 2013; Roostalu et al., 2015; Takeshita et al., 2013). Consequently, these TOG proteins localise to the very MT end, in contrast to EB1 family proteins that bind to an extended region (Maurer et al., 2014). In the absence of tubulin, TOG has been shown to catalyse MT depolymerisation (Brouhard et al., 2008; Roostalu et al., 2015; Shirasu-Hiza et al., 2003).

Fission yeast contains two TOG orthologues, Alp14 (also known as Mtc1) and Dis1 (Garcia et al., 2001; Nakaseko et al., 2001; Ohkura et al., 2001). These two proteins share essential functions; each single deletion mutant is viable, whereas double deletions are inviable (Aoki et al., 2006; Garcia et al., 2002; Hsu and Toda, 2011; Kakui et al., 2013; Nabeshima et al., 1995; Nakaseko et al., 2001). Whereas Alp14 has been shown to be a MT polymerase like other family members (Al-Bassam et al., 2012; Hussmann et al., 2016), a biochemical characterisation of the catalytic properties of Dis1 at MT ends has not yet been performed.

In this study, we explore the biochemical and physiological interplay between Dis1 and Mal3. Dis1 is a MT polymerase that, different from other TOG proteins, also directly binds to Mal3. In combination, the two proteins promote a synergistic change in MT dynamics. Intriguingly, Dis1 does not have a canonical EB1-binding motif. Instead, crystallographic analysis unveiled a non-canonical binding mode, whereby a new motif in Dis1 bound to the conserved hydrophobic cavity in the EBH domain present in Mal3; these structural data demonstrate similarities and differences in the interaction between EB1 and the SXIP motif versus that used by the unconventional EB1-binding motif in Dis1. Genetic studies demonstrate that the interaction between Dis1 and Mal3 is of physiological significance.

## RESULTS

### Fission yeast Dis1 is a MT polymerase

**Fig. 1.**
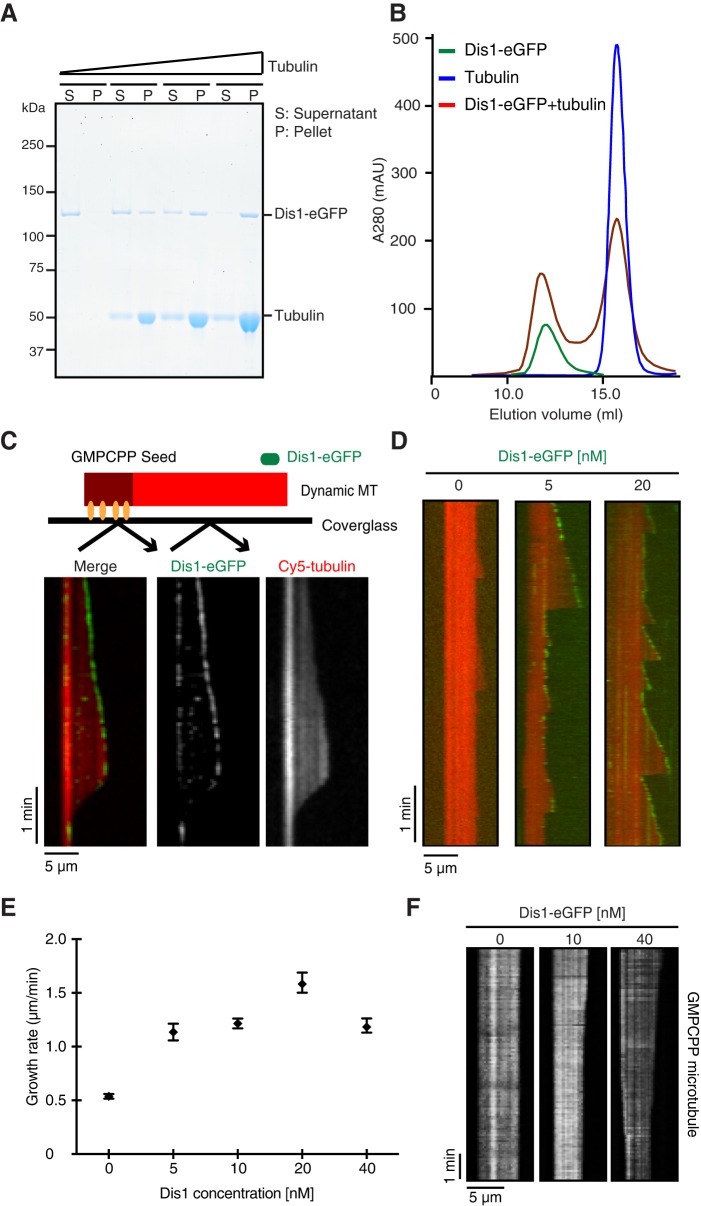
**Fission yeast Dis1 is a MT polymerase.** (A) The MT-binding activity of Dis1–eGFP. Dis1–eGFP concentrations were kept constant at 0.2 µM, while the tubulin concentration was varied (from left to right, 0, 1, 2 and 4 µM). Both supernatant (S) and pellet (P) were stained with Coomassie Brilliant Blue. (B) Tubulin-binding activity of Dis1–eGFP. 5 µM Dis1–eGFP (green), 10 µM tubulin dimers (blue) and their mixture (red) were fractionated on gel filtration columns. (C) Schematic of the assay (top) and dual-colour TIRF-M kymographs showing binding events between 10 nM Dis1–eGFP (green in merge) and the ends of growing MTs. (D) Dual-colour TIRF-M kymographs showing Cy5-labelled MTs growing in the absence or presence of Dis1–eGFP (green). (E) Plot of the mean growth rate. The Cy5-labelled tubulin concentration was 8 µM in C–E. Data points, black; error bars are s.e.m., *n*=50. (F) Kymographs showing GMPCPP-stabilised Cy5-labelled MTs. Scale bars: 5 µm (horizontal) and 1 min (vertical) (C,D,F).

We bacterially expressed and purified recombinant full-length Dis1 protein with a hemagglutinin (HA) tag or eGFP fused to its C-terminus (Fig. S1A). It has been demonstrated that the ability of XMAP215 to accelerate MT polymerisation is dependent on its interaction with both soluble tubulin and MTs (Widlund et al., 2011). Like XMAP215, purified Dis1–eGFP co-sedimented with paclitaxel (taxol)-stabilised MTs (Fig. 1A) and also formed stable complexes with soluble tubulin dimers (Fig. 1B; Fig. S1B). These data indicate that Dis1 shares the binding characteristics of a MT polymerase.

To observe how Dis1–eGFP localises on dynamic MTs and how it affects their dynamic properties, we performed a total internal reflection fluorescence microscopy (TIRF-M)-based *in vitro* assay (Fig. 1C and Movie 1). MTs were grown from immobilised and GMPCPP-stabilised MT seeds (Materials and Methods) in the presence of Cy5-labelled tubulin and GTP (Bieling et al., 2010). Dis1–eGFP associated weakly with the MT lattice and accumulated on and tracked growing MT plus-ends in a spot-like manner (Fig. 1C), similar to other TOG orthologues (Brouhard et al., 2008; Li et al., 2012; Podolski et al., 2014); by contrast, Dis1–eGFP was not observed on shrinking MT ends, similar to Alp14, the other TOG orthologue in fission yeast (Al-Bassam et al., 2012; Garcia et al., 2001; Hussmann et al., 2016; Nakaseko et al., 2001).

Next, we measured the effect of Dis1 on the dynamics of individual MTs (Fig. 1D). MT dynamic instability parameters, such as the average growth rate, shrinkage rate and catastrophe frequency, were quantified for different Dis1 concentrations (Table S1; Fig. 1E). The strongest polymerase effect was measured for a concentration of 20 nM Dis1, where it accelerated the growth of MTs by approximately threefold, from 0.54±0.02 µm/min to 1.58±0.09 µm/min (mean±s.e.m., *n*=50; *P*<0.0001 by a Student's *t*-test) (Fig. 1E). By contrast, Dis1 did not have a strong impact on the shrinkage rate or catastrophe frequency (Fig. S1C). Interestingly, similar to XMAP215 and ch-TOG (Brouhard et al., 2008; Roostalu et al., 2015), Dis1 also facilitated the depolymerisation of GMPCPP-stabilised MTs in the absence of free tubulin (Fig. 1F; Fig. S1D). These data show that Dis1 is a bona fide MT polymerase.

### Dis1 directly binds to Mal3 and, synergistically, they alter the dynamic properties of MTs

**Fig. 2.**
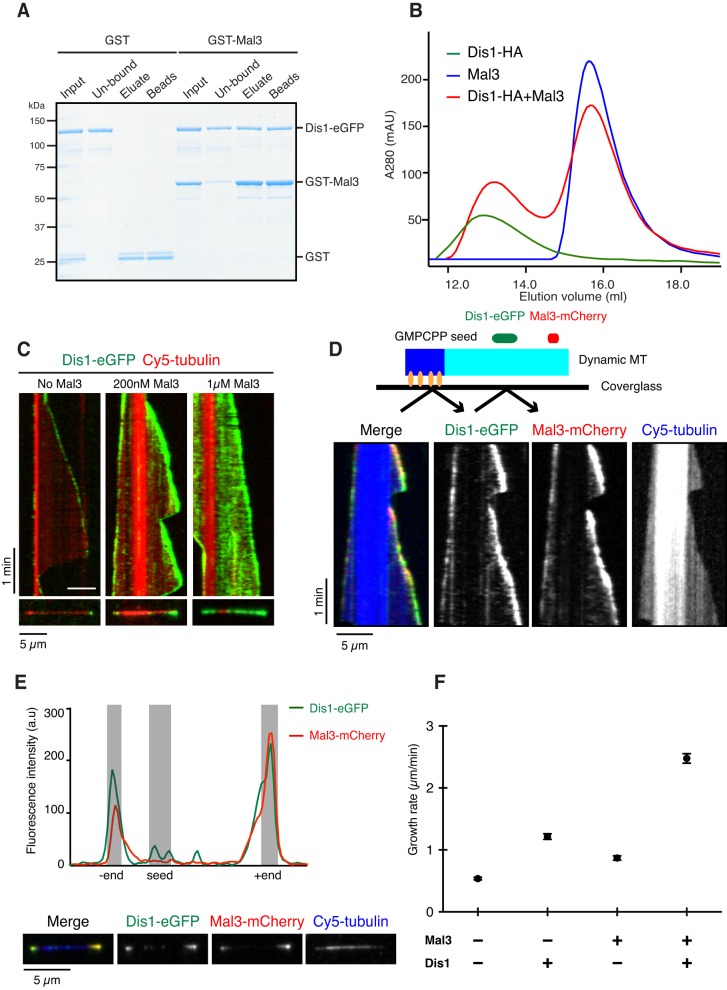
**Dis1 directly binds to Mal3.** (A) Binding between Dis1 and Mal3. GST or GST–Mal3 bound to glutathione beads was mixed with Dis1–eGFP. (B) Analytical gel filtration chromatography. 5 µM Dis1–HA (green), 20 µM Mal3 (blue) or the mixture of both proteins (red) was analysed. (C) TIRF-M kymographs showing binding events between 20 nM Dis1–eGFP (green) and the ends of growing Cy5-labelled MTs (red) in the absence or presence of unlabelled Mal3. The Cy5-labelled tubulin concentration was 20 µM. Snapshots are shown at the bottom. (D) Schematic of the assay (top) and triple-colour TIRF-M kymographs showing binding events between 10 nM Dis1–eGFP (green in merge) and the ends of growing Cy5-labelled MTs (blue in merge) in the presence of 200 nM Mal3–mCherry (red in merge) (bottom). (E) 10 nM Dis1–eGFP accumulates at the ends of a MT in a similar manner to 200 nM Mal3–mCherry. The Cy5-labelled tubulin concentration was 10 µM in D and E. Scale bars: 5 µm (horizontal) and 1 min (vertical) (C–E). (F) Plot of the mean growth rate. 10 nM Dis1 and/or 20 nM Mal3 were added in the presence of 8 µM tubulin. Data points, black; error bars are s.e.m., *n*=50.

Previous studies have shown evidence for a functional interplay between TOG and EB1 proteins (Li et al., 2012; van der Vaart et al., 2011; Zanic et al., 2013), although there are no reports showing a direct interaction between these two +TIPs. In fission yeast, the Alp14 localisation to MT plus-ends is independent of Mal3 (Al-Bassam et al., 2012); whether this is also the case for Dis1 has not been tested. Hence, we bacterially expressed and purified recombinant full-length Mal3 proteins with or without eGFP or mCherry fused to its C-terminus (Fig. S2A). We found that GST-tagged Mal3 (GST–Mal3), but not GST alone, pulled down Dis1–eGFP (Fig. 2A). Furthermore, analytical gel filtration demonstrated that recombinant Dis1 with an HA tag (Dis1–HA) and Mal3 form a stable complex in solution (Fig. 2B; Fig. S2B). These data show that Dis1 directly binds to Mal3.

Next, we examined the effect of Mal3 on the localisation of Dis1–eGFP on dynamic MTs using our TIRF-M assay (Fig. 2C). Whereas 20 nM Dis1–eGFP localised to growing MT ends in a spot-like manner, as with XMAP215 (Brouhard et al., 2008; Li et al., 2012; Podolski et al., 2014) (Fig. 2C and Movie 2), upon the addition of 200 nM Mal3, the amount of Dis1–eGFP at MT ends was strongly increased; Dis1–eGFP localised now to a much more elongated region at growing MT ends, rather reminiscent of the appearance of end-tracking Mal3–GFP (Fig. S2C and Movie 3) (Maurer et al., 2011). In the presence of the higher concentrations of 1 µM Mal3, Dis1–eGFP bound to the entire MT lattice (except for the GMPCPP seeds) (Fig. 2C; Movie 4), again similar in appearance to the localisation of Mal3–GFP at this high concentration (Fig. S2C).

To simultaneously visualise the localisation of Dis1, Mal3 and MTs, we used triple-colour TIRF-M. We observed that Dis1–eGFP and Mal3–mCherry colocalised to the MT end region (Fig. 2D,E; Movie 5). In control experiments, we observed that Mal3 did not directly bind to Alp14 (Fig. S2D). These results demonstrate that Mal3 recruits Dis1, but not Alp14, to the extended EB-binding region at growing MT ends.

We then examined the effect of Mal3-dependent Dis1 MT end recruitment on the dynamic properties of MTs. Compared to tubulin alone, 20 nM Mal3 mildly accelerated the growth rate by 1.6-fold (0.54±0.02 µm/min versus 0.87±0.04 µm/min; mean±s.e.m., *n*=50, *P<*0.0001 by a Student's *t*-test). 10 nM Dis1 accelerated the growth rate by 2.3-fold (1.22±0.05 µm/min; mean±s.e.m., *n*=50, *P*<0.0001). In the presence of both 20 nM Mal3 and 10 nM Dis1, the growth rate was even more strongly increased by 4.6 fold (to 2.48±0.08 µm/min; mean±s.e.m., *n*=50, *P*<0.0001), suggesting synergistic action of the two proteins (Table S2, Fig. 2F, Fig. S2E), reminiscent of what has been reported for the vertebrate homologues of these +TIPs (Li et al., 2012; Zanic et al., 2013). In addition, we found that Mal3 and Dis1 together substantially promoted the MT catastrophe frequency (∼3.8-fold); Mal3 alone had a modest impact (∼1.9-fold), whereas Dis1 had no effect on its own (Fig. S2F). It is of note that no synergistic effect was observed for the shrinkage rate (Fig. S2F). Therefore, the increased accumulation of Dis1 at MT ends mediated by Mal3 together with the presence of Mal3 itself strongly enhanced the dynamicity of MTs, in particular increasing the growth rate and the catastrophe frequency. Taking all these data together, we conclude that Mal3 specifically binds to Dis1 but not to Alp14, thereby enhancing MT dynamicity.

### The C-terminal tail region of Dis1 is the primary site for Mal3 binding

**Fig. 3.**
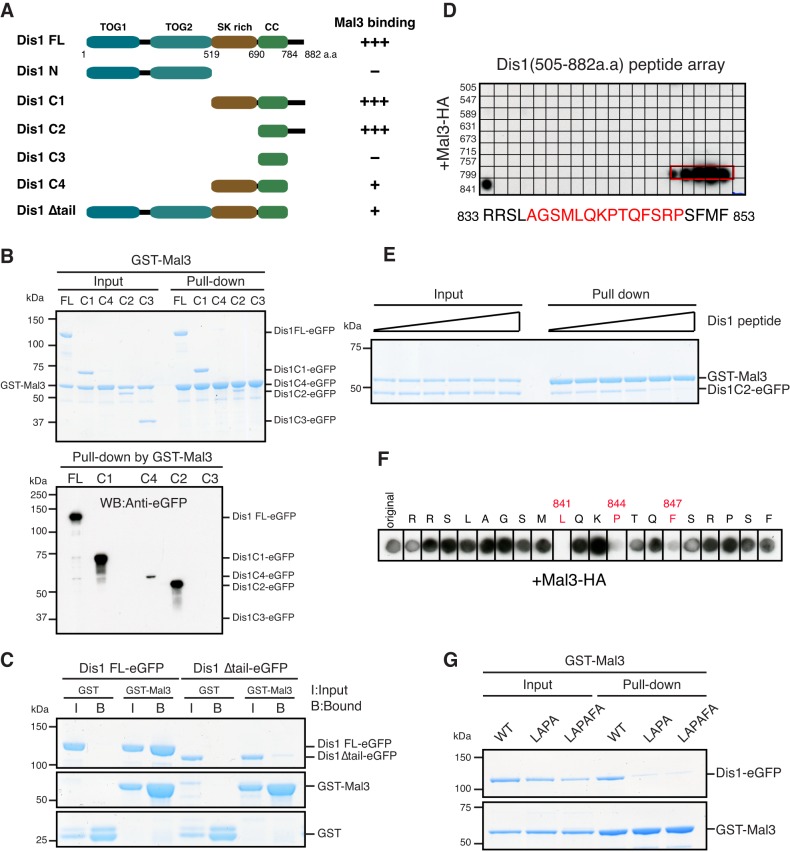
**The C-terminal tail region of Dis1 is the primary binding site for Mal3.** (A) A schematic representation and a summary of their binding to Mal3. TOG1, TOG2, TOG homology domains; SK rich, rich in serine and lysine residues; CC, coiled-coil. (B) Binding between various truncated Dis1 proteins and Mal3. GST–Mal3 bound to glutathione beads was mixed with truncated Dis1–eGFP. The vertical line found between the last two lanes on the right was introduced unintentionally during scanning of the original data. (C) Binding between the full-length or tail-less Dis1 protein and Mal3. (D) Peptide array analysis. The arrays cover from 505th to 882nd amino acid residues of Dis1. The arrays were incubated with a solution containing Mal3–HA protein, followed by immunoblotting against an anti-HA antibody. Amino acid sequences corresponding to bound residues (red) are shown on the bottom. (E) Binding between the tail region of Dis1 and Mal3. GST–Mal3 bound to the glutathione beads were pre-incubated with various concentrations of the Dis1 peptide (amino acids 833–852) (from left to right, 0, 0.5, 2.5, 5.0, 50, 100 µM) for 30 min before incubation with 0.5 µM Dis1 C2–eGFP for an additional hour. (F) Alanine-scanning mutagenesis analysis. Each position in the Dis1 peptide was replaced with alanine. Amino acid residues shown in red were sensitive to alterations. (G) Binding between Mal3 and the wild-type (WT) or mutated Dis1 protein. Dis1–eGFP proteins were LAPA (L841A and P844A) or LAPAFA (L841A, P844A and F847A).

Although Dis1 directly binds to Mal3 (Fig. 2), there is no SXIP motif or CAP-Gly domain within the Dis1 protein. Therefore, we decided to experimentally identify the interaction site of Dis1 for Mal3 binding. Using GST pulldown assays with several truncated Dis1 constructs (Fig. 3A), we found that the C-terminal region of Dis1 (Dis1 C2, amino acids 690–882) is the minimal region that interacts with Mal3 as efficiently as full-length Dis1 (Dis1 FL); the preceding coiled-coil region (Dis1 C3, amino acids 690–784) alone did not bind to Mal3 (Fig. 3A,B). This data indicated that the C-terminal tail of Dis1 (amino acids 784–882) is necessary for Mal3 binding. To test this possibility, we constructed the C-terminally truncated Dis1 that lacked this region (Dis1Δtail) and compared the binding to Mal3 between Dis1 FL and Dis1Δtail. We found that the binding of Dis1Δtail to Mal3 was substantially reduced compared to that of Dis1 FL (Fig. 3A,C).

Consistent with these pulldown assays, TIRF-M assays demonstrated that Dis1 constructs containing the C-terminal tail could track the MT plus-end in the presence of Mal3 (Fig. S3A,B). Importantly, although Dis1 FL could track the MT end regardless of the presence or absence of Mal3, Dis1 C2 (amino acids 690–882), which lacked the N-terminal part of Dis1 required for its autonomous end-tracking ability, tracked ends only in the presence of Mal3 (Fig. S3B). Notably, Dis1 C2 in the presence of Mal3 tracked a more extended region at MT ends compared to Dis1-FL alone, which showed a spot-like appearance on the MT ends. These data strongly suggest that the C-terminal tail region is the primary site for Mal3 binding.

### Three amino acid residues in the C-terminus of Dis1 are crucial for binding to Mal3

To identify the Mal3-binding site within the C-terminal tail region of Dis1, we analysed the Dis1–Mal3 interaction by means of a tiling peptide array. A membrane was spotted with 20-residue peptides covering the C-terminal region of Dis1 (amino acids 505–882) with a two-residue start increment per spot, and then probed with Mal3–HA protein followed by immunoblotting. Remarkably, a single region consisting of 21 amino acid residues (amino acids 833–853) bound to Mal3 (Fig. 3D). To verify this interaction, we synthesised the corresponding peptide (Dis1 peptide) and conducted a competition experiment in the GST pulldown assay. Confirming the importance of the identified peptide for the Dis1–Mal3 interaction, an excess amount of the Dis1 peptide effectively abolished the binding between Mal3 and Dis1 C2 (amino acids 690–882) (Fig. 3E).

To identify the amino acid residues that are most crucial for the interaction between Dis1 and Mal3, we further performed alanine-scanning mutagenesis against each amino acid and found that the Dis1 residues L841, P844 and F847 were indispensable residues for Mal3 binding (Fig. 3F). To further test the importance of these key residues, we mutated them in full-length Dis1; we constructed two Dis1 replacement mutants (LAPA, L841A and P844A; and LAPAFA, L841A, P844A and F847A) and compared their binding to Mal3. Both Dis1 mutant proteins bound more weakly to Mal3 in comparison to wild-type Dis1 (Fig. 3G), confirming that the three amino acid residues L841, P844 and F847 in the Dis1 C-terminal region are crucial for the interaction with Mal3. Given that the identified binding motif differs from the canonical SXIP motif, Dis1 likely binds to Mal3 in a new and unconventional manner.

### The coiled-coil and EBH domains of Mal3 are necessary and sufficient for binding to Dis1

**Fig. 4.**
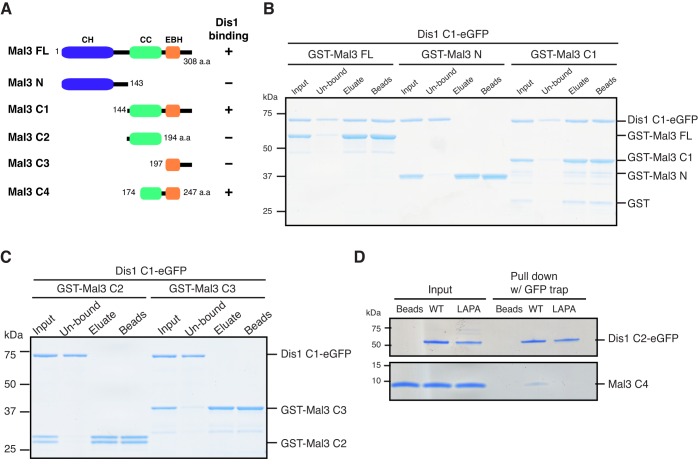
**The coiled-coil and EBH domains of Mal3 are necessary and sufficient for binding to Dis1.** (A) A schematic representation of various truncated Mal3 proteins and a summary of their binding to Dis1. CH, calponin-homology domain; CC, coiled-coil; EBH, EB homology. (B,C) Binding between various truncated Mal3 proteins and Dis1. Dis1 C1 (amino acids 520–882) tagged with eGFP was mixed with various GST-tagged truncated Mal3 proteins (B, FL, N, C1 or C, C2, C3), which were bound to the glutathione beads. (D) Binding between the dimerised EBH domain of Mal3 protein and Dis1. EBH-domain-containing Mal3 protein (amino acids 174–247) was mixed with wild-type (WT) or mutated Dis1-C2–eGFP (LAPA; L841A, P844A) and pulled down by GFP trap beads.

To identify in turn the Dis1-binding region within Mal3, several truncated Mal3 constructs were made and used for Dis1-binding GST pulldown assays. We found that the C-terminal half (Mal3 C1, amino acids 144–308) is responsible for binding to Dis1 (Fig. 4A,B). This C-terminal region consists of the parallel coiled-coil region (amino acids 144–194) followed by the EBH domain (amino acids 197–247) (Slep et al., 2005) (Fig. 4A). We produced fragments corresponding to these two regions (Mal3 C2 and Mal3 C3, respectively) and found that neither bound to Dis1 (Fig. 4C). However, a Mal3 construct that consists of the C-terminal part of the coiled-coil and the EBH domains (Mal3 C4, amino acids 174–247) was sufficient to bind to Dis1 (Fig. 4A,D). Introduction of the LAPA mutations (L841A and P844) to the Dis1 C2 construct abrogated the binding of Mal3 C4 (Fig. 4D). Taken together, the dimerised EBH domain within Mal3 is necessary and sufficient for the binding to Dis1.

### Direct interaction between Dis1 and Mal3 is essential for the synergistic impact on MT dynamics

**Fig. 5.**
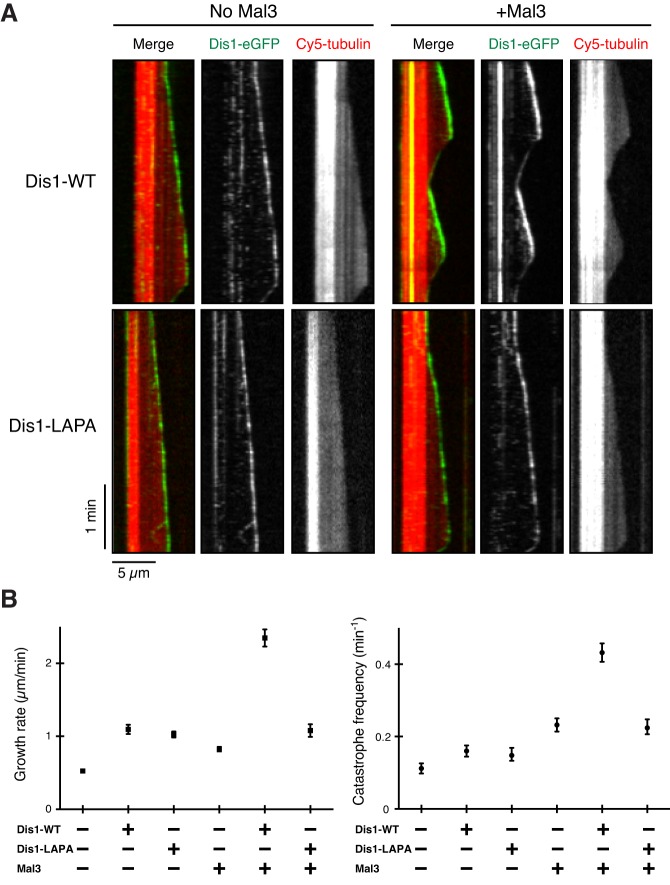
**Physical binding between Dis1 and Mal3 is required for the synergistic impact on MT dynamics.** (A) TIRF-M kymographs showing Dis1–eGFP (10 nM, green in merge) in the presence or absence of 20 nM Mal3. The Cy5-labelled tubulin (red) concentration was 8 µM. Scale bars: 5 µm (horizontal) and 1 min (vertical). (B,C) Plot of the mean growth rate (B) or catastrophe frequency (C). 10 nM Dis1-eGFP [wild-type (WT) or LAPA] and/or 20 nM Mal3 were added in the presence of 8 µM tubulin. Data points, black; error bars are s.e.m. *n*=50.

We next examined the localisation of 10 nM Dis1-LAPA–eGFP in the presence or absence of 20 nM Mal3 on dynamic MTs using TIRF-M assays. As shown in Fig. 5A, the localisation patterns of Dis1-LAPA to the MT tip were indistinguishable between in the presence and in the absence of Mal3; they displayed thin spot-like appearances rather than the thick comets seen in wild-type Dis1–eGFP in the presence of Mal3. This firmly established that Dis1-LAPA fails to interact with Mal3 not only in solution but also on MTs.

We then asked whether Dis1 and Mal3 in combination are capable of altering the dynamic properties of MTs without their direct interaction. To this end, we quantified various parameters of the MT dynamics. Remarkably, no additive or synergistic impact was observed between Dis1-LAPA and Mal3 (Fig. 5B; Table S3); the growth rate was 1.08±0.09 µm/min (mean±s.e.m., *n*=50), which was very similar to that of Dis1-LAPA alone (1.02±0.04 µm/min, mean±s.e.m.), whereas the catastrophe frequency was almost identical to that of Mal3 alone (0.22±0.02 µm/min versus 0.23±0.01 µm/min; mean±s.e.m., *n*=50). It is of note that authentic activities of Dis1-LAPA on its own towards the MT dynamics are indistinguishable to those of wild-type Dis1 (Fig. 5B; Table S3). Collectively, these results illuminate the marked differences in the TOG and EB1 families between fission yeast and vertebrates; whereas fission yeast Dis1 (the TOG protein) and Mal3 (the EB1 protein) need to interact directly in order to exert a synergistic impact on the MT dynamics, vertebrate orthologues are poised to do so without physical binding.

### Crystallographic analysis of the Mal3–Dis1 binding interface

**Fig. 6.**
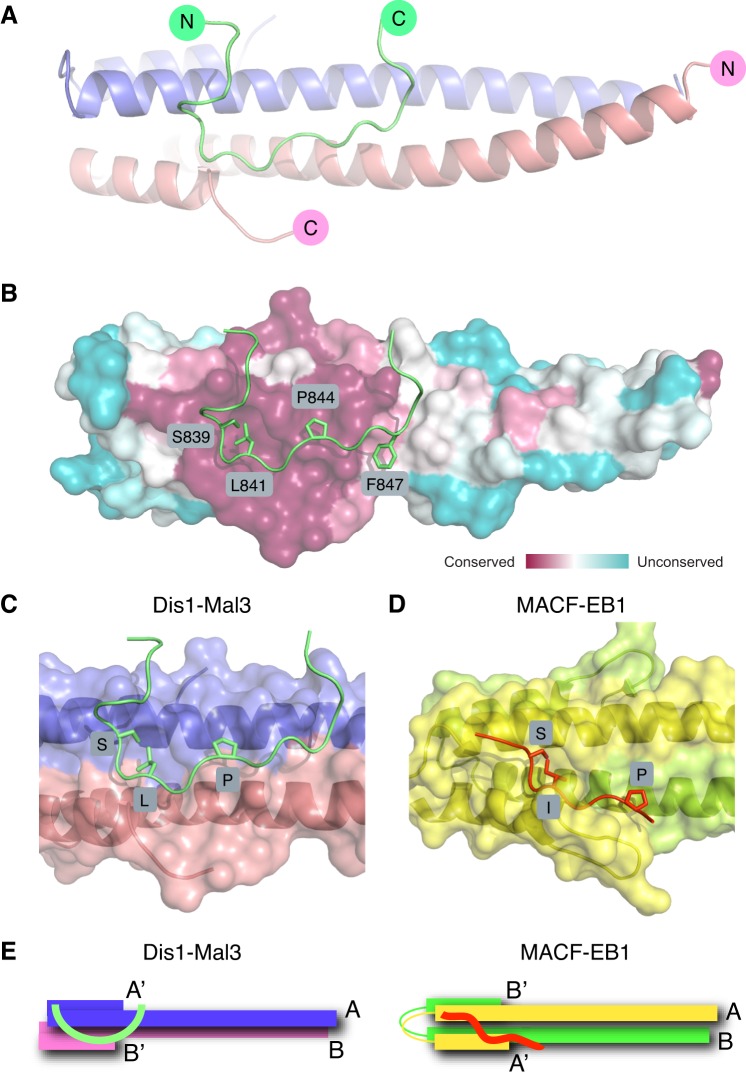
**Molecular details of the Mal3–Dis1 interaction.** (A) Ribbon diagram of the complex between the coiled-coil and EBH domains of Mal3 (residues 174–247 forming a homodimer as depicted in blue and pink) and the interacting Dis1 peptide (residues 833–852 as depicted in green). A second peptide binds on the other side of the dimer, but has been omitted from the figure for the sake of clarity. (B) Details of the key interacting residues. Surface conservation plot of the EBH domain of Mal3 from low (blue) to high (purple) similarity. (C,D) Close-up view of the interaction seen between the Mal3-EBH dimer (residues 174–247 depicted in blue and pink) and the Dis1 peptide (residues 833–852 depicted in green) (C) or the human EB1-EBH dimer (residues 191–260 depicted in yellow and green) and the SXIP motif containing the MACF peptide (residues 5468–5497 depicted in orange) (D). (E) Cartoon showing the comparison of the interaction interfaces between Mal3–Dis1 (left) and EB1–MCAF (right). A,A′ and B,B′ represent the EBH domain derived from two dimerised Mal3 molecules.

Previous crystal structures of the EB1 C-terminal domain showed that the highly conserved surface of the EBH domain comprises a hydrophobic cavity, which is important for the binding to SXIP-containing +TIPs (Honnappa et al., 2005, 2009; Slep et al., 2005). A crystal structure of the corresponding Mal3 EBH domain has not been reported yet. To obtain structural insight into the basis of Mal3–Dis1 interaction, we crystallised Mal3 C4 (amino acids 174–247) alone and in complex with the minimal Mal3-binding Dis1 peptide (amino acids 833–852) (Fig. 3E), and solved their structures (Fig. 6A; Fig. S4A,B and Table S4). Like the human EBH domain (Honnappa et al., 2005; Slep et al., 2005), the homodimerised EBH domain of Mal3 also displayed a hydrophobic cavity, demonstrating that this structural feature is conserved (Fig. 6B). The Dis1 peptide bound to this homodimerised EBH domain (Fig. 6A; Fig. S4A,B), and its binding was predominantly driven by hydrophobic interactions between L841, P844 and F847 in the Dis1 peptide and the conserved hydrophobic cavity on the outside edge of the EBH domain of Mal3 (Fig. 6B). These structural data are fully consistent with the results of alanine scanning and pull-down assay using the mutant Dis1 protein with the disrupted binding motif (Fig. 3F,G).

Next, we carried out a detailed comparison of the interaction interface between Mal3–Dis1 and EB1–MACF (microtubule actin cross-linking factor), an EB1 interactor containing the canonical SXIP motif (Honnappa et al., 2009) (Fig. 6C,D; Fig. S4C,D). We found that the S^839^MLQKP^844^ residues in the Dis1 peptide are functionally equivalent to the S^5477^LIPTP^5482^ motif in the MACF peptide, especially the L841 residue in the Dis1 peptide and the corresponding I5479 residue in the MACF peptide, which fit into the conserved hydrophobic cavity formed by the EBH domain. Intriguingly, however, we noted a clear difference between the individual interaction modes of the two peptides: whereas the binding interface for EB1–MCAF was provided predominantly by one EB1 monomer, the interface for the Dis1 peptide spans two Mal3 monomers (Fig. 6E). This structural data explains the necessity of dimerisation of the EBH domain for Dis1 binding through the preceding coiled-coil of Mal3 (Fig. 4A,C). These findings indicate that the binding between Dis1 and Mal3 is indeed non-canonical, although the hydrophobic cavity is conserved between Mal3 and EB1.

### Physiological significance of the binding between Dis1 and Mal3

**Fig. 7.**
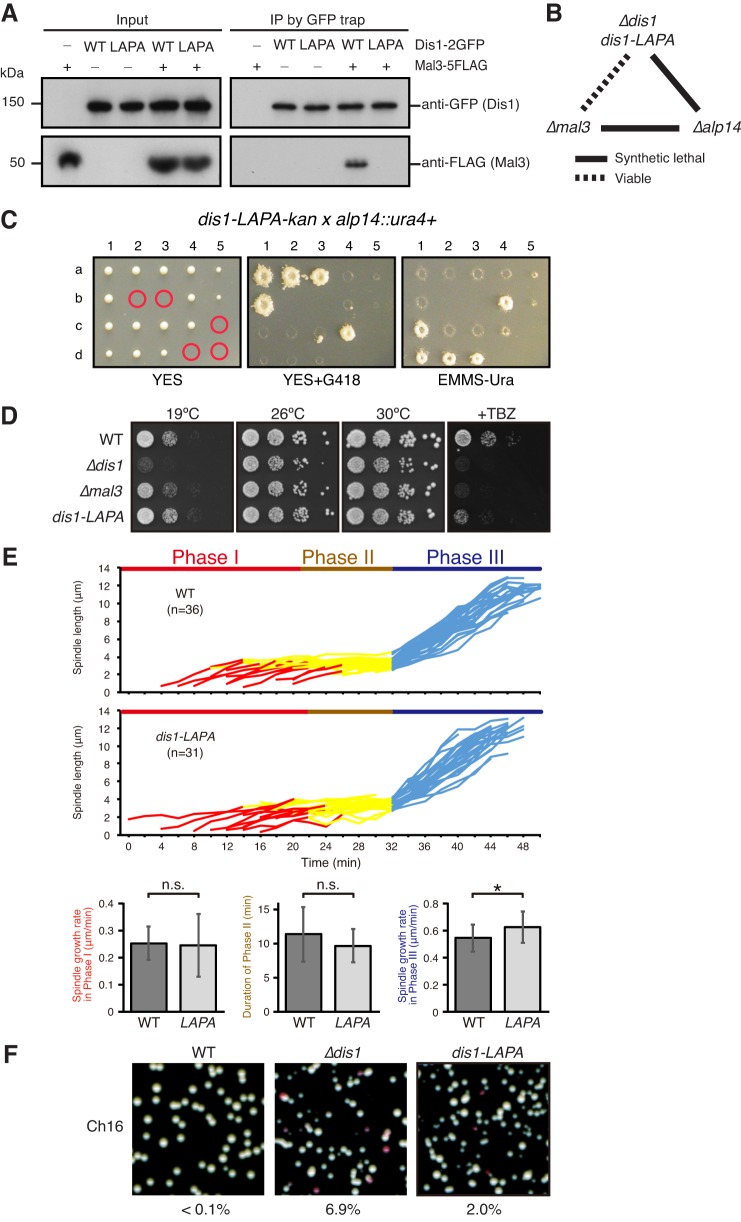
**Dis1 interacts with Mal3 *in vivo* and its interaction is crucial for Dis1 function.** (A) Interaction between Dis1 and Mal3. Whole-cell extracts (1.0 mg) were pulled down with GFP trap beads. Immunoblotting was performed with anti-GFP and anti-FLAG antibodies. Input: 15 µg. WT, wild type. (B) Summary of genetic interaction. Solid lines, synthetically lethal; dotted line, viable. (C) Tetrad dissection. Five representative tetrads are shown [the parental ditype (1), tetratypes (2–4) and the non-parental ditype (5)]. Double mutants were inviable (red circles). (D) Spot assay. Indicated strains were serially diluted (tenfold each), spotted on YES plates and incubated for 4 days or at 26°C for 4 days in the presence of 10 µg/ml TBZ (right). (E) Mitotic progression and MT dynamics in *dis1-LAPA* cells. Mitotic progression of wild-type (left, *n*=38) and *dis1-LAPA* (right, *n*=31) cells containing a tubulin marker (mCherry–Atb2) was followed in live cells under fluorescence microscopy at room temperature (23°C). Phase I (red), the initial stage of spindle elongation; phase II (yellow), the pre-anaphase stage with constant spindle length; Phase III (blue), anaphase B (Nabeshima et al., 1998). Results are mean±s.d.; *n*>30 cells; *P<0.01; n.s., not significant (two-tailed unpaired Student's *t*-tests). (F) Minichromosome loss assay. Indicated strains carrying the minichromosome Ch16 (Niwa et al., 1989) were grown on rich YE plates (lacking adenine) and incubated at 25°C for 6 days. The percentage of red and/or sectored colonies is shown at the bottom (*n*≥1,000).

Given that Dis1 and Mal3 directly interact *in vitro*, we next examined their *in vivo* interaction with GFP trap pulldown from fission yeast lysates. We found that Dis1–2GFP and Mal3–5FLAG indeed formed a complex (Fig. 7A). We then created a mutant strain in which wild-type *dis1^+^-2GFP* was replaced by *dis1-LAPA-2GFP* (L841A and P844A). Consistent with the *in vitro* data (Fig. 3F,G), the pulldown assay clearly indicated that Dis1-LAPA–2GFP did not bind to Mal3–5FLAG (Fig. 7A), demonstrating that the identified Mal3-binding site in Dis1 is relevant in the context of living cells.

In order to explore the functional significance of the direct binding between Dis1 and Mal3, we examined genetic interactions. It has been previously shown that the double mutant between Δ*dis1* and Δ*alp14* was synthetically lethal (Garcia et al., 2002; Nakaseko et al., 2001). Interestingly, whereas the Δ*mal3* mutant displayed synthetic lethality with Δ*alp14*, Δ*mal3*Δ*dis1* was viable (Kerres et al., 2004)*.* This result implies that Dis1 and Mal3 act in the same genetic pathway, which shares essentiality with Alp14 (Fig. 7B). Moreover, *dis1-LAPA* mutants also showed synthetic lethal interaction with Δ*alp14* (Fig. 7B,C). It is noteworthy that, unlike Δ*dis1* cells, which displayed cold sensitivity (Ohkura et al., 1988), *dis1-LAPA* cells were capable of forming colonies at low temperature (Fig. 7D). This result indicates that failure of interaction between Dis1 and Mal3 did not abolish all the cellular functions of Dis1 (Garcia et al., 2002; George and Walworth, 2015; Sanchez-Perez et al., 2005); instead by forming a complex with Mal3, Dis1 plays an essential role in cell division, which can be compensated for by Alp14.

Although a strain containing *dis1-LAPA* cells grew apparently normally, these cells displayed hyper-sensitivity to the MT-destabilising drug thiabendazole (TBZ) (Fig. 7D), which slows down growth in mutants with defects in MT regulation and chromosome segregation (Garcia et al., 2001; Toyoda et al., 2002; Umesono et al., 1983); note that Δ*mal3* and Δ*dis1* cells were also individually hypersensitive to TBZ (Fig. 7D) as previously shown (Aoki et al., 2006; Beinhauer et al., 1997). We then examined MT integrity and mitotic progression in the *dis1-LAPA* mutant. No noticeable differences in MT structures and intensities were observed; however, a modest yet reproducible increase in the rate of MT elongation during anaphase B (phase III, Nabeshima et al., 1998) was detected (Fig. 7E). Currently the reason for this faster anaphase B progression is unknown; nonetheless, this result uncovered that a direct interaction between Dis1 and Mal3 plays a role in the proper MT dynamics in late mitosis.

Mutations in genes required for chromosome transmission fidelity can be identified by a minichromosome loss assay (Fleig et al., 1996; Niwa et al., 1989; Takahashi et al., 1994). It was previously observed that both *mal3* and *dis1* mutants display a minichromosome loss phenotype (Aoki et al., 2006; Beinhauer et al., 1997). Although it was not as severe as in Δ*dis1* cells (6.9%), a strain containing Dis1-LAPA lost minichromosomes at an elevated rate compared to wild-type cells (2.0% versus <0.1%) (Fig. 7F). Taken together, these results indicate that the physical interaction between Dis1 and Mal3 is indeed necessary for accurate chromosome segregation.

## DISCUSSION

Our study of the biochemical and physiological interplay between fission yeast Dis1 and Mal3 has provided five major results. First, Dis1 is a bona fide MT polymerase like other TOG proteins. Second, Dis1 and Mal3 synergistically control MT dynamics. Third, this is explained by a direct interaction between Dis1 and Mal3 involving a new EB1-binding motif. Fourth, the binding mode of this motif in Dis1 to the conserved hydrophobic pocket in the EBH domain of Mal3 is unconventional. Fifth and, finally, the Dis1–Mal3 interaction is of physiological significance.

Our *in vitro* reconstitution experiments showed that Dis1 is a MT polymerase that autonomously localises to the growing MT plus-ends, thereby accelerating the MT growth rate by about threefold. This behaviour is very similar to that of Alp14, the other TOG orthologue in fission yeast (Al-Bassam et al., 2012; Garcia et al., 2001; Nakaseko et al., 2001). As for other TOG proteins, such as frog XMAP215, budding yeast Stu2 or fission yeast Alp14 (Al-Bassam et al., 2006, 2012; Hussmann et al., 2016; Widlund et al., 2011), the N-terminal TOG domains of Dis1 are likely to bind tubulin dimers, whereas the central Ser-Lys-rich (SK-rich) domain of Dis1 is probably responsible for binding to the MT lattice, which is consistent with our results with truncated Dis1–eGFP constructs (Fig. S3B) and also with the detailed domain analysis previously performed (Nakaseko et al., 1996).

The vertebrate proteins *Xenopus* XMAP215 and human EB1 have previously been reported to synergistically increase the MT polymerisation rate *in vitro*, although these two proteins do not interact directly with each other (Zanic et al., 2013). Synergy was proposed to result from the ability of EB1 to potentially alter the curvature of the MT end structure allosterically, allowing XMAP215 to promote MT growth more efficiently (Zanic et al., 2013). Here, we have also observed a synergistic enhancement of the MT growth rate by fission yeast Mal3 and Dis1, even if to a lesser extent. For the fission yeast proteins, synergy is likely to arise from the enhanced accumulation of Dis1 at MT ends as a consequence of its recruitment by Mal3 through direct interaction, as our TIRF microscopy experiments have demonstrated. Consistent with this notion, the Dis1 mutant protein with specific defects in binding to Mal3 (Dis1-LAPA) failed to exhibit additive or synergistic effects on the MT dynamics in combination with Mal3, although Dis1-LAPA on its own still retained normal enzymatic activities (Fig. 5B). In this context, it is interesting to note that in budding yeast, an interaction between Bim1 (an EB1 protein) and Stu2 (a TOG protein) is known; however their mode of interaction is unclear (Wolyniak et al., 2006). This suggests that fungi and vertebrates have developed similar yet distinct regulatory systems by which to regulate the MT dynamics changed mediated by the TOG and EB1 families.

**Fig. 8.**
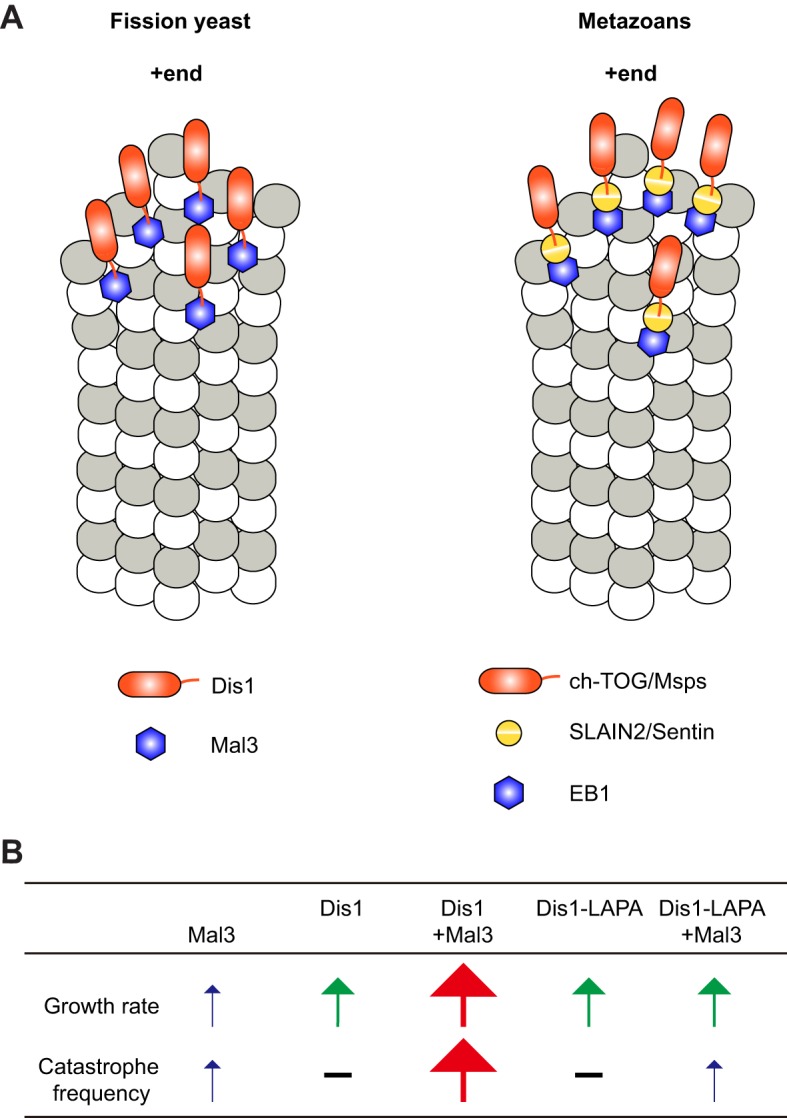
**Interplay between Dis1 and Mal3 and comparison to metazoans.** (A) In fission yeast, Dis1 (orange) binds directly to Mal3 (blue), thereby enhancing the MT growth rate and the catastrophe frequency in a synergistic manner (left). By contrast, in metazoans including humans and *Drosophila*, ch-TOG and Msps (orange) interact with EB1 (blue) through SLAIN2 and Sentin (yellow), respectively (right), thereby accelerating MT dynamics. Dimer formation of EB1/Mal3 is not shown for simplicity. (B) Summary of individual parameters representing MT dynamics in the presence of Dis1 (wild-type or LAPA) and/or Mal3. –, not altered compared to control samples (tubulin only); blue arrow, increased by up to twofold; green arrow, increased by ∼threefold; red arrow, increased by more than fourfold.

In human cells, the protein SLAIN2 acts as a linker mediating an indirect interaction between ch-TOG and EB1 (van der Vaart et al., 2011). The effect of all three human proteins together on MT dynamics has not been investigated yet *in vitro*. However, the indirect interaction between XMAP215 and EB1 proteins is probably conserved in metazoa, as the unrelated *Drosophila* protein Sentin plays a similar role for *Drosophila* Msps (a TOG protein) and EB1, mediating their indirect interaction (Gluszek et al., 2015; Mohan et al., 2013; Moriwaki and Goshima, 2016; Li et al., 2011, 2012). A mild synergistic effect on the growth rate of MTs exerted by a combination of these three *Drosophila* proteins has also been observed, possibly as a consequence of their tripartite interaction (Li et al., 2012). Taken together, it is possible that members of the TOG and EB1 families act collaboratively at the MT plus-ends in many, if not all, eukaryotes, having a synergistic impact on MT dynamics. Their interaction can either be indirect as in metazoans, or direct as in fission yeast, demonstrating evolutionary plasticity of this regulatory module (Fig. 8A,B).

Here, we have solved the first X-ray structure of the EBH domain of Mal3, the protein originally used to reconstitute autonomous MT end tracking by an EB protein and its recruitment of other +TIPs to growing MT ends (Bieling et al., 2007). Our crystallographic analysis shows the two hydrophobic pockets that are formed by the C-terminal part of the Mal3 EBH domains and to which typically recruited +TIPs bind through their canonical SXIP motif. This pocket is conserved, in agreement with the observation of the SXIP motif containing proteins being recruited by Mal3 *in vitro* (Bieling et al., 2007). The polypeptide fold, however, is unconventional, because a hydrophobic pocket is formed from helices of two Mal3 polypeptides (trans-configuration), whereas in EB1 a hydrophobic pocket is formed by the corresponding helices of a single EB1 polypeptide (cis*-*configuration).

The interaction of the non-canonical Dis1 peptide with the hydrophobic pocket is also unconventional compared to the binding of SXIP-motif-containing peptides. Although both peptides interact strongly with the hydrophobic cavity of the EBH domain, their exact binding interface varies (Fig. 6E). Owing to these differences, the binding interface in Mal3–Dis1 is at 90° to the linker between the two helices in the EB monomer, whereas in EB1–MACF (containing the canonical SXIP motif) it is on the same side (Fig. 6C–E; Fig. S4C,D). Hence, the SXIP motif is not be the sole motif that can interact with the EBH domain of EB1. In fact, previous comprehensive proteomic analysis of EB1-binding proteins identified a fairly large number of interactors that do not contain the canonical SXIP sequence (Jiang et al., 2012; Tamura et al., 2015). Therefore, it is likely that non-canonical EB1 interaction motifs similar to the one we discovered in Dis1 will exist in proteins of other organisms than fission yeast, likely also including humans.

Previous work has shown that Dis1 localises to the mitotic kinetochore through interaction with the Ndc80 (also known as Hec1) outer kinetochore component and that it plays a crucial role in proper kinetochore–MT attachment (Hsu and Toda, 2011). It is, therefore, likely that Dis1 and Mal3 interact at the kinetochore–MT interface upon kinetochore capture by the MT plus-ends. Interestingly, Alp14 also localises to the mitotic kinetochore, yet in a different manner; Alp7 (a TACC family protein) binds to Ndc80, thereby recruiting the Alp7–Alp14 complex to the kinetochore (Sato et al., 2004; Tang et al., 2015). The result that *Δalp14* and Mal3-binding defective *dis1-LAPA* display synthetic lethality indicates that these two fission yeast TOG members in concert establish and maintain kinetochore–MT attachment by exploiting distinct binding partners, Mal3 for Dis1 and Alp7 for Alp14.

Intriguingly, the interaction between TOG and the Ndc80 complex is conserved in budding yeast and humans (Miller et al., 2016). Importantly, TOG orthologues of these two organisms (Stu2 and ch-TOG) regulate kinetochore–MT attachment in a tension-sensitive manner. Intriguingly, *in vitro* work showed that the TOG proteins play this crucial role without altering MT dynamics (Miller et al., 2016). However, in cells undergoing mitosis, the fine-tuning of MT dynamics and the state of tension should temporally be coupled. We envisage that the EB1 proteins might be important proteins promoting proper kinetochore–MT attachment in response to the state of tension. Given these circumstances, *in vitro* studies examining addition of the EB1 proteins (and SLAIN2) to TOG and the Ndc80 complex would be of great interest to explore this possibility.

In conclusion, our work provides new insight into the unconventional mode through which Mal3 binds with Dis1, which is likely to indicate the possibility of further modes of interactions for other uncharacterised EB1 interactors. From an evolutionary point of view, the interaction between EB1 and TOG proteins is probably ubiquitous; intriguingly, however, the mechanism by which they interact varies among different organisms, illuminating diversification and remarkable adaptability through evolution.

## MATERIALS AND METHODS

### Protein expression and purification

*dis1* cDNA optimised for *E. coli* codons was synthesised (GeneArt Gene Synthesis) and subcloned into the pETM-11 vector (Maurer et al., 2011). The purification of the various Dis1–eGFP constructs was performed as described previously (Maurer et al., 2011) except that Dis1 buffer [50 mM Tris-HCl pH 7.5, 300 mM KCl, 10% (v/v) glycerol, 1 mM EDTA and 1 mM DTT] containing EDTA-free protease inhibitors (Roche) and IgG Sepharose 6 Fast Flow (GE Healthcare) were used. In addition, TEV protease (for Dis1–eGFP) or Precision protease (for Dis1–HA) was used. The purification of the Mal3, Mal3–eGFP and Mal3–mCherry was performed as described previously (Maurer et al., 2011).

The EBH domain (amino acids 174–247) of Mal3 was purified by the same procedures as described above except that buffer A (50 mM Tris-HCl pH7.5, 500 mM NaCl and 10 mM β-mercaptoethanol) containing 30 mM imidazole was used for cell suspension and column washing. In addition, His-tag purification resin (Roche) and buffer A supplemented with 500 mM imidazole were used for affinity purification and elution, respectively. The cleavage of the oligo-histidine tag was performed by incubation with TEV protease as described earlier. Mal3 proteins were further purified by gel filtration using Superose200 10/300 columns equilibrated with buffer B (20 mM Tris-HCl pH 7.5, 100 mM NaCl, 2 mM DTT).

Porcine brain tubulin was purified and cycled tubulin fractions were labelled with Cy5 (Lumiprobe) or EZ-link NHS Biotin (Thermo Scientific) by standard methods as described previously (Bieling et al., 2007). The protein concentration was determined by Bradford assay (Bio-Rad).

### MT co-sedimentation and binding assays

The MT co-sedimentation assay was performed as described previously (Maurer et al., 2011). 0.2 µM Dis1–eGFP was incubated with taxol-stabilised MTs at concentrations from 0 to 4 µM in BRB80 supplemented with 85 mM KCl and 85 mM CH3COOK in the presence of 20 µM taxol.

### Analytical gel filtration

5 µM Dis1–eGFP and/or 10 µM tubulin (or 5 µM Dis1–HA and/or 20 µM Mal3) was incubated in gel filtration buffer (25 mM K-HEPES pH 7.5, 200 mM KCl, 1 mM EGTA, 1 mM MgCl_2_) before loading on a Superose 6 10/300 (GE Healthcare) equilibrated with gel filtration buffer. The absorbance of the eluted protein was measure at 280 nm.

### TIRF microscopy and image analysis

TIRF-microscopy-based dynamic MT assays were performed as previously described (Bieling et al., 2010). TIRF assay buffer consisted of BRB80 supplemented with 85 mM KCl, 1 mM GTP, 10 mM β-mercaptoethanol, 0.1% Brij-35, 0.1% methylcellulose (Sigma-Aldrich) and an oxygen scavenger system [glucose, glucose oxidase (Serva) and catalase (Sigma-Aldrich)]. For simultaneous dual (or triple)-colour time-lapse imaging of the Cy5 and GFP (and mCherry) channel, imaging was performed at 1-s intervals with 100 ms exposure time, using a ×100 objective lens at 30±1°C on a custom TIRF microscope equipped with a Cascade II, cooled charge-coupled device camera (Photometrics), illuminating the sample with 488 nm and 640 nm (and 561 nm) lasers. Image analysis was performed as described previously (Duellberg et al., 2014).

### GST pulldown assay

For domain analysis of the Dis1 protein, 1 µM of GST or GST–Mal3 and 0.5 µM of several truncated Dis1–eGFP proteins were incubated in pulldown buffer (BRB80 supplemented with 85 mM KCl, 0.01% Brij-35 and 1 mM DTT). Then, 20 µl of glutathione–Sepharose beads (GE Healthcare) were added and after incubation for 1 h at 4°C, beads were washed and eluted with the buffer containing 10 mM reduced glutathione. For domain analysis of Mal3, 0.5 µM Dis1 C1-eGFP and 1 µM of several truncated GST–Mal3 proteins were used and the same procedures were followed.

For the peptide competition assay, 1 µM of purified GST–Mal3 bound to the glutathione beads were pre-incubated with various concentration of the Dis1 peptide (amino acids 833–852; 0, 0.5, 2.5, 5.0, 50, 100 µM) in pulldown buffer for 30 min, followed by further incubation with 0.5 µM Dis1 C2-eGFP for 1 h.

### Peptide array assay

Peptide array assays were performed as previously described (Hsu and Toda, 2011).

The blocked membrane was incubated with 1 µg/ml Mal3–HA protein and bound Mal3–HA was detected by immunoblotting with an anti-HA antibody (diluted 1:1000, 16B12, Covance).

### X-ray crystallography

X-ray data were collected on beamlines I04 and I03 of the Diamond Light Source. Data were integrated and scaled using the Xia2 pipeline. The unliganded Mal3 EBH (hereafter Mal3) domain was solved using *ab initio* molecular replacement as implemented in the ARCIMBOLDO-LIGHT package (Sammito et al., 2015). 20-residue α-helical segments were used as a search model. A strong (30.2% origin) native Patterson peak at 0.155, 0.5, 0.5 in the native data suggested the presence of translational pseudo-symmetry, and the appropriate correction was applied in Phaser. The preliminary poly-alanine model was then assigned sequence in Buccaneer, and manually rebuilt and refined with Coot (Emsley and Cowtan, 2004) and Refmac5 (Murshudov et al., 2011). The final model was refined with anisotropic temperature factors applied to all atoms. The Mal3–Dis1 structure was solved by molecular replacement in Phaser (McCoy et al., 2007) using the apo-Mal3 structure as a search model. Model building and refinement were carried out as for the apo-structure. Clear density could be seen for the bound Dis1 peptides in an unbiased difference map at both homodimer interfaces and was built in the final stages of refinement. All data collection and refinement statistics are provided in Table S4.

### Crystallisation and structure solution

Crystals were grown by sitting-drop vapour diffusion. The Mal3^174-247^ proteins at 7 mg/ml were mixed with crystallisation solution comprising 0.2 M NaCl, 30% MPD and 0.1 M sodium acetate at pH 4.6. Crystals grew to full size after 5 days and were flash-cooled in liquid nitrogen with no additional cryoprotection. To obtain the Mal3 EBH domain-Dis1 complex, the Mal3^174-247^ protein was incubated with the Dis1 peptide (residues 833-852) in a 1:5 molar ration for 1 h at 4°C before crystallisation. Crystals were grown by sitting-drop vapour diffusion. The protein complex at 7 mg/ml was mixed with the crystallisation solution consisting of 0.2 M MgCl_2_, 0.1 M HEPES pH 7, 20% PEG 6K. Crystals grew to full size after a week and were flash-cooled in liquid nitrogen.

### Immunoprecipitation

Immunoprecipitation was performed as previously described (Hsu and Toda, 2011) except that immunoprecipitation buffer [50 mM Na-HEPES, pH7.5, 150 mM NaCl, 1 mM EDTA, 0.1% Tween20, 1 mM DTT, protease inhibitor cocktail (Sigma-Aldrich)], GFP-Trap A and an anti-FLAG antibody (diluted 1:2000, Anti-FLAG M2, Sigma-Aldrich) or an anti-GFP antibody (diluted 1:2000, Anti-eGFP 632569, Clontech) was used.

### Fission yeast strains, media, genetic methods, minichromosome loss assay and cell biology

Fission yeast strains used in this study are listed in Table S5. Standard fission yeast methodologies were followed (Moreno et al., 1991). Spot assays were carried out by spotting 5–10 μl of cells at a concentration of 2×10^6^ cells/ml after 10-fold serial dilutions onto rich YE5S plates with or without a drug (10 µg/ml TBZ). C-terminal tagging and gene disruption were performed using PCR-generated fragments as described previously (Bähler et al., 1998; Sato et al., 2005). The minichromosome loss assay was carried out as described previously (Niwa et al., 1989; Tange et al., 2016).

Fluorescence microscope images were obtained using the DeltaVision microscope system (Applied Precision, Inc.) with a cooled CCD camera CoolSNAP.HQ (Photometrics). Live cells were imaged in a glass-bottomed culture dish (MatTek Corporation) coated with soybean lectin.

### Statistical data analysis

In all the experiments in which statistical data are presented, the number of experiments and sample size are described in the main text, tables and figure legends. Results are expressed as the mean±s.e.m. or mean±s.d. Statistical evaluation was performed using Prism (GraphPad). All *P*-values are from two-tailed unpaired Student *t*-tests. We followed this key for asterisk placeholders for *P*-values in the figures: *P<0.01, n.s. not significant.

## Note added in proof

After acceptance of this manuscript, a new paper on the interaction between budding yeast Kar9 and Bim1 (the yeast EB1) was published (Manatschal et al., 2016). In this work, it is shown that the amino acid sequence containing TRLRPPTPLS binds to Bim1. We notice that ‘TRLRPPTPLS’ is very similar to ‘SMLQKPTQFS’ included in the Dis1 peptide. It is possible that Kar9 binds to Bim1 in a non-canonical manner analogous to the binding between Dis1 and Mal3.
